# Recruitment of Fkh1 to replication origins requires precisely positioned Fkh1/2 binding sites and concurrent assembly of the pre-replicative complex

**DOI:** 10.1371/journal.pgen.1006588

**Published:** 2017-01-31

**Authors:** Allan Reinapae, Kristiina Jalakas, Nikita Avvakumov, Marko Lõoke, Kersti Kristjuhan, Arnold Kristjuhan

**Affiliations:** Department of Cell Biology, Institute of Molecular and Cell Biology, University of Tartu, Tartu, Estonia; National Institute of Environmental Health Sciences, UNITED STATES

## Abstract

In budding yeast, activation of many DNA replication origins is regulated by their chromatin environment, whereas others fire in early S phase regardless of their chromosomal location. Several location-independent origins contain at least two divergently oriented binding sites for Forkhead (Fkh) transcription factors in close proximity to their ARS consensus sequence. To explore whether recruitment of Forkhead proteins to replication origins is dependent on the spatial arrangement of Fkh1/2 binding sites, we changed the spacing and orientation of the sites in early replication origins *ARS305* and *ARS607*. We followed recruitment of the Fkh1 protein to origins by chromatin immunoprecipitation and tested the ability of these origins to fire in early S phase. Our results demonstrate that precise spatial and directional arrangement of Fkh1/2 sites is crucial for efficient binding of the Fkh1 protein and for early firing of the origins. We also show that recruitment of Fkh1 to the origins depends on formation of the pre-replicative complex (pre-RC) and loading of the Mcm2-7 helicase, indicating that the origins are regulated by cooperative action of Fkh1 and the pre-RC. These results reveal that DNA binding of Forkhead factors does not depend merely on the presence of its binding sites but on their precise arrangement and is strongly influenced by other protein complexes in the vicinity.

## Author summary

In this study, we explore the mechanisms that determine activation of DNA replication origins in early S phase. It has been shown that a subset of replication origins is regulated by Forkhead family transcription factors that ensure their firing at the beginning of S phase. However, the recruitment of Forkhead factors to replication origins is not a straightforward process–there are thousands of Forkhead binding sites in the genome and their presence does not guarantee that Forkheads actually bind these sites. We show that recruitment of Fkh1 protein to DNA replication origins requires precise arrangement of Forkhead binding sites and depends on formation of pre-replicative complexes at the origins. These results clarify the mechanisms of Forkhead-dependent regulation of early DNA replication origins and also reveal that mere presence of consensus binding sites is not sufficient for recruitment of Forkhead proteins to their target loci.

## Introduction

Replication of genomic DNA in budding yeast (*Saccharomyces cerevisiae*) is initiated from hundreds of origins throughout the S phase. Replication origins can be characterized by their efficiency, which refers to the probability that a particular origin will fire in a given cell cycle, and by the timing of their firing in the S phase. In general, early firing origins are also efficient, i.e. replication is initiated from these origins in almost every S phase. However, the determinants of early origin firing are not fully understood. It has been shown that origins located in euchromatic regions close to centromeres fire early in the S phase, while origins found in sub-telomeric heterochromatin are generally late-firing [1, 2]. Relocation of several origins into ectopic loci has revealed that some origins adjust their firing time according to the local chromatin context, while a set of chromosomal localisation-independent origins retain their early-firing pattern in the new location [3].

Both origin relocations and genome-wide DNA replication initiation studies have shown that Forkhead transcription factor family members Fkh1 and Fkh2 are required to ensure early firing of chromosomal localisation-independent origins. These findings were further confirmed by the fact that these origins contain at least two consensus binding sites for Fkh1/2 proteins, as well as by observations that disruption of these sites leads to a loss of the origin’s early firing signature [3, 4]. The consensus binding sequence for Forkhead family proteins, RYMAAYA, is rather loosely defined and allows many variations in the actual DNA sequence [5]. Therefore, approximately 46,000 Fkh1/2 consensus sequences can be found throughout the budding yeast genome, but only about 1650 of them are actually bound by Forkhead factors [6]. Remarkably, overexpression of Fkh1 leads to its recruitment to multiple new loci that were not occupied at the normal expression level of Fkh1 [7], indicating that availability of free Forkhead proteins might be limiting in cells. This also suggests that in the presence of great excess of potential binding sites the Forkheads are stably recruited only to the loci that either are more accessible, or where their binding is supported by other proteins. It is not clear how the binding of Fkh proteins is regulated in replication origins. However, mutation of one of the two Fkh sites present at *ARS305*, *ARS607* and *ARS737* significantly reduces binding of Fkh1 to these origins [3]. This suggests that multiple Fkh1/2 consensus sites in close proximity to each other are required for efficient binding of Fkh1. In addition, due to the asymmetrical nature of the Fkh1/2 consensus binding sequence, the efficient binding of Forkhead factors may be influenced by orientation of the sites. Interestingly, three chromatin-independent early-firing replication origins *ARS305*, *ARS607* and *ARS737*, contain two Fkh1/2 sites in divergent orientation relative to each other and separated by 72 base pairs, suggesting that the overall configuration of the Forkhead binding motifs at early replication origins is carefully conserved.

To elucidate the role of the precise orientation and spacing of Fkh1/2 sites in regulation of early-firing replication origins, we tested the efficiency of Fkh1 binding to origins with altered patterns of Fkh1/2 consensus sites, as well as the firing profile of such origins. We show that only wild-type configuration of Forkhead binding sites leads to efficient Fkh1 recruitment to an origin in G1 and to early firing of the origin in the S phase. We also demonstrate that even when the consensus sites are present in their wild-type configuration, Fkh1 fails bind to the origin if recruitment of the Mcm2-7 complex is disrupted. These results suggest that efficient DNA binding of Forkhead family proteins is strongly influenced by cooperative interactions with other DNA binding factors and is not determined merely by their consensus DNA binding sequence.

## Results

### Proper distance between Fkh1/2 sites is required for Fkh1 binding and early firing of *ARS607*

**Fig 1:**
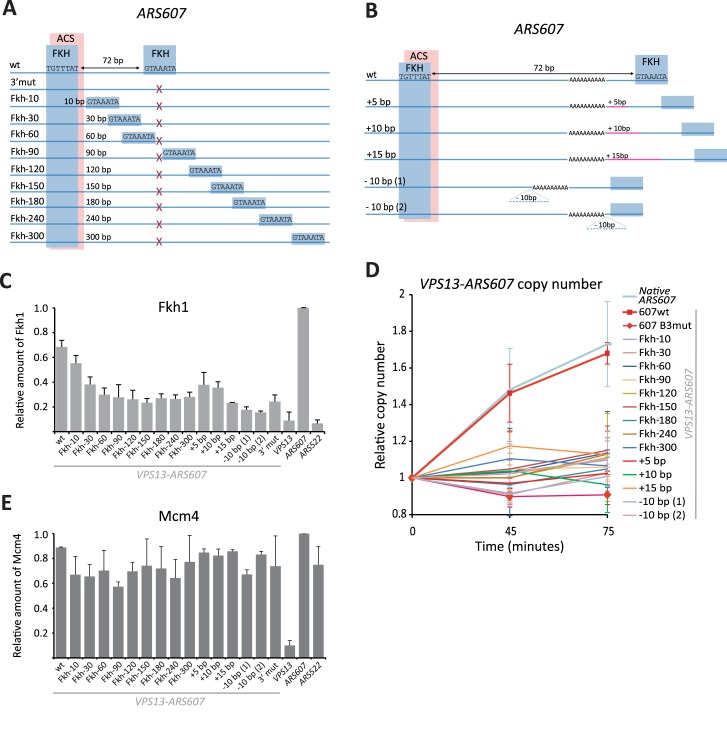
Alterations of distance between the Fkh1/2 binding sites in *ARS607*. **(A)** Schematic representation of *ARS607* origins inserted into the *GAL*-*VPS13* locus. Approximate locations of Fkh1/2 consensus binding sites (blue boxes) and the ACS (pink box) are indicated. In all constructs except wt *ARS607* the ACS-distal (3’) Fkh1/2 binding site was mutated (red X) and a new Fkh1/2 site was introduced at various distances from the ACS-proximal (5’) site. **(B)** Depiction of modified *ARS607* origins with small insertions and deletions between Fkh1/2 binding sites. Approximate locations of Fkh1/2 consensus binding sites (blue boxes), the ACS (pink box), poly-A track and sites of nucleotide insertions or deletions are indicated. Detailed sequences of all modified origin loci are shown in S4 Fig. **(C)** Fkh1 binding to *ARS607* with altered distances between Fkh1/2 sites as determined by ChIP assay. Fkh1 occupancy at mutant *ARS607* is shown relative to its binding to the native *ARS607* locus in the same strain. A strain with no ARS in the *VPS13* locus and the native late-replicating origin *ARS522* that contains no Fkh1/2 binding sites are shown as controls (*VPS13* and *ARS522*, respectively). **(D)** Relative copy number of *VPS13*-*ARS607* DNA in HU-arrested cells. Cells were arrested in G1 with α-factor and then released into HU-containing media for 45 and 75 minutes. Graphs show the ratio of *VPS13*-*ARS607* and late-replicating *ARS522* loci, the ratio in G1-arrested cells was set as 1. Relative copy number of the native *ARS607* locus is shown as control. Full data for each strain is shown in S1 Fig. **(E)** Mcm4 binding to *ARS607* loci with altered distances between Fkh1/2 sites as determined by ChIP assay. Mcm4 occupancy at mutant *ARS607* is shown relative to its binding to the native *ARS607* origin in the same strain. A strain with no ARS in the *VPS13* locus and the native origin *ARS522* are shown as controls (*VPS13* and *ARS522*, respectively).

Our previous study has revealed that two Fkh1/2 binding sites were required for early firing of replication origins *ARS305*, *ARS607* and *ARS737* [3]. At all these origins, one of the Fkh1/2 binding sites is located close to the ARS consensus sequence (ACS) and another is found 72 base pairs away. To test whether the distance between the Fkh1/2 sites is important for efficient binding of Fkh1 and for origin regulation, we made a panel of yeast strains with modified *ARS607* origins inserted into a *GAL-VPS13* locus. In all modified *ARS607* sequences, the native ACS-proximal Fkh1/2 site (partially overlapping with ACS) was left undisturbed, while the distal Fkh1/2 site was mutated (Fig 1A). Mutation of the distal consensus sequence led to a significant drop in Fkh1 occupancy, although it remained higher than at loci that do not contain any Fkh1/2 binding sites (Fig 1C). We then introduced a new Fkh1/2 site at various distances from the proximal site and determined the efficiency of Fkh1 binding to these origins in G1-arrested cells. When the Fkh1/2 sites were separated by 10 base pairs, binding of Fkh1 protein to the origin was detected at slightly reduced levels compared to the wild-type (wt) sequence (Fig 1C). In contrast, binding of Fkh1 to all other origins was indistinguishable from that seen at an origin where only a single Fkh1/2 site was present, suggesting that precise distance between Fkh1/2 binding sites in *ARS607* is critical for efficient recruitment of Fkh1 protein to the origin (Fig 1C). To finely probe the tolerance of Fkh binding to altered size of the gap between Fkh1/2 sites, we made 5 bp, 10 bp and 15 bp insertions and two different 10 bp deletions between the Fkh1/2 binding sites in *GAL-VPS13-ARS607* (Fig 1B and S4 Fig). In these constructs, modifications of *ARS607* were minimal and importantly, all modified loci retained the original Fkh1/2 sites in their immediate surrounding sequences. This minimized the possibility that recruitment of Fkh1 was affected by local DNA sequence context rather than by the change in gap size between the Fkh1/2 sites. We found that all introduced modifications, even insertion of 5 bp into the locus, caused significant drop of Fkh1 binding to the origin (Fig 1C).

Concordant with their impaired ability to bind the Fkh1 protein, origins *ARS305*, *ARS607* and *ARS737* with mutated Fkh1/2 binding sites also lose their early firing pattern *in vivo* [3]. To determine whether the distance of Fkh1/2 sites in *ARS607* is crucial also for early firing of the origin, we arrested cells in G1 and released them synchronously into S phase in the presence of hydroxyurea (HU), thus enabling the firing of early but not late origins. The dynamics of the relative copy number of the *GAL-VPS13-ARS607* locus following the release from G1 block were determined by qPCR analysis of extracted genomic DNA. If the origin could fire early in S phase, the locus initiated replication in the presence of HU and the relative amount of its DNA was expected to increase during the experiment. This assay revealed that only the strain with wt *ARS607* was able to support early replication of the *GAL-VPS13-ARS607*, while in all other strains the locus was not replicated in the presence of HU (Fig 1D and S1 Fig). Interestingly, the origin where the two Fkh1/2 sites were separated by 10 bp did not support early replication of the locus, despite the fact that Fkh1 occupancy at that origin was relatively high (Fig 1C). This suggests that the proper spacing of Fkh1/2 binding sites rather than the mere binding of Fkh1 is critical for the early firing of the origin. To show that the alterations in *ARS607* sequence that affected its early firing did not render it inactive, we confirmed that the Mcm2-7 complex was recruited to all modified *GAL-VPS13-ARS607* loci, indicating their proper licensing (Fig 1E).

### Divergent orientation of Fkh1/2 sites is required for Fkh1 binding to replication origins

**Fig 2:**
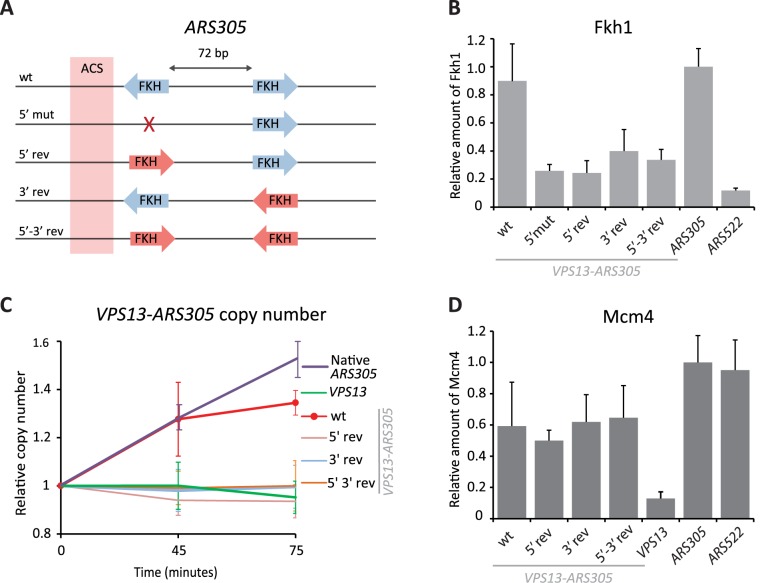
Alterations of Fkh1/2 site orientations in *ARS305*. **(A)** Schematic representation of *ARS305* origins inserted into the *GAL*-*VPS13* locus. Approximate locations of Fkh1/2 consensus binding sites (blue arrows) and the ACS (pink box) are indicated. In 5’mut construct, the ACS-proximal Fkh1/2 site was disrupted by mutation (red X). In Fkh1/2 site reversal mutants, the sites were inverted relative to their original orientations (red arrows). **(B)** Fkh1 binding to *ARS305* loci with various orientations of Fkh1/2 sites as determined by ChIP assay. Fkh1 occupancy to *ARS305* mutants is shown relative to its binding to the native *ARS305* locus. Fkh1 binding to the native late-replicating origin *ARS522* is shown as control (*ARS522*). **(C)** Relative copy number of *VPS13*-*ARS305* DNA in HU-arrested cells. Cells were arrested in G1 with α-factor and then released into HU-containing media for 45 and 75 minutes. Graphs show the ratio of *VPS13*-*ARS305* and late-replicating *ARS522* loci, the ratio in G1-arrested cells was set as 1. Relative copy number of the native *ARS305* and origin-free *VPS13* loci are shown as controls. Full data for each strain is shown in S2 Fig. **(D)** Mcm4 binding to *ARS305* loci with various orientations of Fkh1/2 sites as determined by ChIP assay. Mcm4 occupancy to mutant *ARS305* is shown relative to its binding to the native *ARS305* locus. The strain with no ARS in the *VPS13* locus and the native origin *ARS522* are shown as controls (*VPS13* and *ARS522*, respectively).

In early firing origins *ARS305*, *ARS607*, and *ARS737* the asymmetrical Fkh1/2 consensus sequences are found in divergent orientation, suggesting that directionality, or symmetry of Fkh1/2 sites may be important for efficient binding of Forkhead factors to replication origins. To test this hypothesis, we reversed the orientations of Fkh1/2 consensus sites in *ARS305* (Fig 2A) and tested whether this affected the efficiency of Fkh1 binding and early firing of the origin. Fkh1 binding was nearly lost in all Fkh1/2 binding site reversal mutants, including the one in which both sites were rotated to form a convergent conformation, implying that the proper orientation of Fkh1/2 sites is critical for Fkh1 binding to *ARS305* (Fig 2B). As expected, none of the above mutants were able to fire early in S phase when cells were released into HU-containing media (Fig 2C and S2 Fig), although all of them were properly licensed (Fig 2D).

### Divergently oriented Fkh1/2 binding sites are enriched in early replication origins

**Fig 3:**
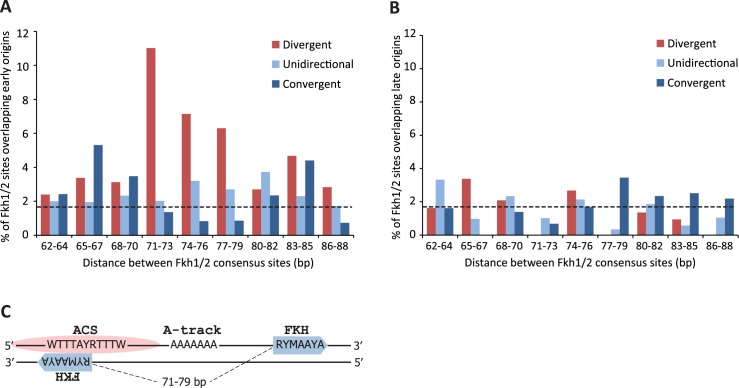
Analysis of Fkh1/2 binding sites at replication origins. **(A)** Double Fkh1/2 binding sites (RYMAAYA) with different spacing and orientation were located throughout the yeast genome. The sites were then mapped onto a genome-wide early DNA replication initiation profile [8]. Percentage of double Fkh1/2 sites that co-localize with early-replicating loci is shown. Black dashed line indicates the background and is calculated as average frequency of overlap between scrambled Fkh1/2 double consensus sites (in all orientations; with 50–100 bp gap) and early origins. **(B)** Distribution of double Fkh1/2 sites at late replication origins was analysed as in **A**. **(C)** The general pattern of sequence elements in early replication origins with divergent Fkh1/2 binding sites. Two Fkh1/2 sites (blue arrows) are separated by a stretch of 71–79 bp linker DNA that contains a poly-A track. Location of the ACS (pink ellipse) varies, but is typically found in close proximity to or overlapping with one Fkh1/2 site. The ACS-proximal Fkh1/2 consensus sequence is located on the DNA strand complementary to that containing the ACS. The model is based on alignment of 20 early origins containing divergent Fkh1/2 binding sites (see text and S3 Fig for details).

As the Forkhead consensus sequence RYMAAYA allows numerous variations in the actual DNA sequence, these sites can be found frequently throughout the genome. Our results with modified origins *ARS607* and *ARS305* suggest that the two Fkh1/2 sites must be present in proper orientation and with correct spacing between them for Forkhead-dependent regulation of these origins. To find out how many double Fkh1/2 sites are present in the yeast genome and how many of them co-localize with early replication origins, we searched the yeast genome for locations of Fkh1/2 consensus binding sites and plotted those onto a genome-wide early DNA replication initiation profile, based on BrdU incorporation into DNA in the presence of HU [8]. Specifically, we sought loci where two Fkh1/2 consensus sites were separated by 62–88 base pairs and oriented in three different patterns–divergently, convergently, or unidirectionally. After analyzing the entire yeast genome, we found 5023 double Fkh1/2 sites that were separated by 62 to 88 bp and oriented in one of the three possible patterns (S1 Table). Approximately 3% of these patterns co-localized with early replication origins. However, when both the distance and the orientation of sites were taken into account, the sites in divergent orientation separated by 71–79 bp were almost three times more likely to co-localize with early origins than sites with alternate configurations (Fig 3A). When similar analysis was performed using late replication origins, no overrepresentation of any pattern of Fkh1/2 sites was found. Moreover, divergently oriented sites separated by 71–79 bp were visibly underrepresented in late origins (Fig 3B).

Next, we mapped the locations of ARS consensus sequences in early origins that overlapped with divergent Fkh1/2 sites separated by 71–79 bp. The ACS was found in close proximity (up to 100 bp) to Forkhead binding sites at 20 origins, and alignment of these revealed several common features (Fig 3C and S3 Fig): First, there was no clear preference for position of the ACS within the Fkh motif–it could be found between or outside of the two sites; however, very often ACS partially overlapped with one of the Forkhead consensus sequences. Secondly, the ACS and its proximal Fkh1/2 consensus site were located on complementary strands, i.e. ARS consensus on the T-rich strand and the proximal Forkhead site on the A-rich strand. Thirdly, continuous adenine nucleotide tracks were present between the Fkh1/2 sites. All analysed origins were found to contain either at least one continuous A-track of at least 5 bases, or multiple 4-bp A-tracks. We speculate that these sequences facilitate initial melting of DNA strands during origin activation.

### Origin licensing is a prerequisite for Fkh1 binding to origins

**Fig 4:**
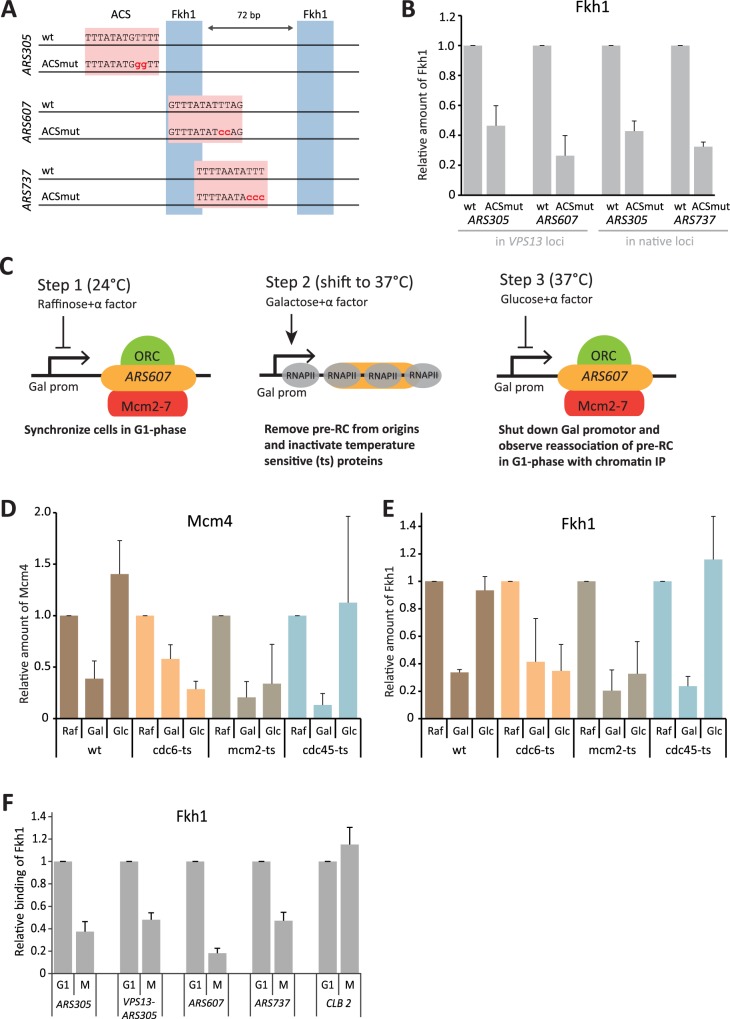
Fkh1 binds to licensed origins. **(A)** Schematic representation of ACS mutants of origins *ARS305*, *ARS607* and *ARS737*. The ACS and Fkh1/2 binding sites are shown as pink and blue boxes, respectively. Wild-type and mutated ACS sequences are shown, with the mutated nucleotides indicated in red. **(B)** Fkh1 binding to ACS-mutated origins. Fkh1 binding to *VPS13*-*ARS* loci in strains carrying wt or ACS-mutated versions of origins were determined by ChIP assay. In all cases, the signals from the strains with wt origins were set to 1. **(C)** Schematic representation of the origin re-licensing assay. Cells with *ARS607* inserted into the *GAL*-*VPS13* locus together with different ts mutations of pre-RC proteins (*cdc6*, *mcm2* and *cdc45*) were grown at a permissive temperature (24°C) and arrested in G1 with α-factor. Arrested cells were transferred to a non-permissive temperature (37°C) and transcription of the *GAL-VPS13* locus was induced by switching to growth medium containing galactose. After two hours, transcription was shut down by transferring the cells into glucose-containing medium to initiate the relicensing of the *ARS607* replication origin. Samples were collected 40 minutes later and the presence of Fkh1 and Mcm4 proteins at the origin was determined by ChIP assay. Binding of Mcm4 **(D)**, or Fkh1 **(E)** to the *GAL*-*VPS13*-*ARS607* locus in *cdc6-ts*, *mcm2-ts* and *cdc45-ts* strains determined by ChIP assay. Samples were taken from G1 arrested cells before transcription induction (Raf), during active transcription (Gal) and after repression of transcription of the locus (Glc). Protein occupancy was normalized to the signal obtained from G1 arrested cells growing at permissive temperature. **(F)** Cell cycle dependent binding of Fkh1 to origins. Cells were first arrested in G1 with α-factor, released to S phase and arrested again in M with nocodazole. Fkh1 binding was determined by ChIP assay and its binding to designated loci in G1 phase was set to 1. Replication-unrelated binding of Fkh1 to *CLB2* promoter was used as a control.

As Fkh1/2 sites are located very close to the ACS in early replicating origins, we tested whether the formation of the pre-RC influences the efficiency of Fkh1 recruitment to these loci. We mutated the ACS sites in *VPS13-ARS305* and *VPS13-ARS607*, as well as in native *ARS305* and *ARS737* loci (Fig 4A), and measured Fkh1 binding to these origins. Although the sequence, spacing and orientation of Fkh1/2 sites were unchanged, the binding of Fkh1 was nearly lost in all ACS-mutated loci (Fig 4B). This indicates that Fkh1 does not bind non-functional replication origins and suggests that recruitment of Fkh1 to early origins may be coupled with origin licensing.

Licensing of replication origins begins with the recruitment of ORC to the ACS motif and is completed during G1 phase by Cdc6- and Cdt1-mediated loading of MCM double hexamer complex onto the origins [9, 10]. In addition, several early firing origins are also pre-loaded with the Cdc45 protein that is required during the subsequent S phase for activation of MCM helicase [11, 12]. To determine which step of origin licensing is necessary for efficient binding of Fkh1 to origins, we monitored its recruitment to the *GAL-VPS13-ARS607* locus in a re-licensing assay in a set of strains expressing temperature sensitive mutants of Cdc6, Mcm2 or Cdc45 proteins. The re-licensing assay using *GAL-VPS13-ARS607* is based on the fact that all pre-RC components can be removed by active transcription over the replication origin and, if the cell remains continuously arrested in G1, these origins become re-licensed rapidly upon shut-down of transcription [13]. The general outline of the assay is shown in Fig 4C. First, cells were arrested in G1 and kept arrested throughout the rest of the experiment. Next, transcription of *GAL-VPS13-ARS607* was activated, leading to displacement of all pre-RC components and Fkh1 proteins from the locus. At the same time, the cells were shifted to a non-permissive temperature to inactivate Cdc6, Mcm2, or Cdc45 proteins. After two hours of incubation, transcription of *GAL-VPS13-ARS607* was repressed, which in turn enabled re-licensing of the locus up to the step where the temperature-sensitive component of the pathway was required. As expected, MCM was reloaded to the locus in wt and *cdc45-ts* strains, but not in *cdc6-ts* and *mcm2-ts* strains (Fig 4D). Similarly, Fkh1 failed to rebind the origin in *cdc6-ts* and *mcm2-ts* strains, while it was efficiently re-recruited in wt and *cdc45-ts* strains (Fig 4E), indicating that Fkh1 was recruited to origins during replication licensing together with or shortly after the loading of MCM complex. These results also suggest that Fkh1 binds replication origins in cell cycle dependent manner, i.e. in G1 when pre-RCs are present, but not in G2/M phase when DNA replication is finished and new pre-RCs are not formed yet. To confirm this, we compared the recruitment of Fkh1 to origins in G1 and M phases of the cell cycle. As expected from earlier results, Fkh1 was bound to *ARS305*, *ARS607*, *ARS737 and VPS13-ARS305* in G1, but not in M phase, while Fkh1 binding to its recognition sequence in the *CLB2* promoter was not affected by the cell cycle (Fig 4F).

## Discussion

In eukaryotic cells, DNA replication is initiated from numerous origins throughout the S phase. Temporal activation of origins is regulated by a variety of factors including the origin’s location and local chromatin context. However, some origins appear to be immune to effects of local chromatin structures and fire early even when transposed to new naturally late-replicating genomic loci. Recent studies have shown that Forkhead family transcription factors are responsible for early activation of many origins. Forkhead-regulated origins typically contain multiple Fkh1/2 consensus binding sites near the ACS, and at least two of these are required for their early activation [3, 4]. Interestingly, at origins where Fkh1 binding was studied in greater detail (*ARS305*, *ARS607* and *ARS737*), the consensus sequences are arranged in an identical pattern: the two sites are oriented divergently and separated by 72 base pairs. Additionally, one of the sites is located very close to the ACS, overlapping it at *ARS607* and *ARS737* (Fig 4A and S3 Fig).

In order to determine whether the exact spacing and orientation of Fkh1/2 sites are critical for Fkh1 recruitment and early activation of these origins, we constructed a panel of yeast strains with altered Fkh1/2 consensus site configuration. At *ARS607*, we altered the spacing between the two sites, while at *ARS305* we modified their relative orientation. Consensus site reversal could not be done in *ARS607* as the proximal site overlaps the ACS and its rotation would have inactivated the origin. On the other hand, multiple Fkh1/2 consensus sites present near the *ARS305* locus left very limited possibilities to change the distance between the two key sites at this origin. By contrast, *ARS607* has no other Fkh1/2 sites within 400 bp of the ACS in 3’ direction. To avoid the possible influence of other DNA replication origins and Fkh1/2 consensus sites near their native loci, the modified *ARS305* and *ARS607* were inserted into the ectopic naturally late-replicating *GAL-VPS13* locus. We have shown previously that both origins are fully functional and fire early in *GAL-VPS13* if their two Fkh1/2 consensus sites remain intact [3, 13].

Our results show that Fkh1/2 sites are very precisely arranged near the early replication origins and that no alterations in their configuration are tolerated. Changing the spacing between the sites or reversing their orientation leads to significant decreases in Fkh1 binding to the origins (Fig 1C and 2B). Accordingly, such modified origins also fail to fire early in the S phase (Fig 1D and 2C). The only exception to this general pattern was observed when the distance between the two sites was reduced to 10 bp. However, while this change had only a modest effect on Fkh1 binding, the altered origin failed to fire early in S phase. This observation underscores the delicate nature of the mechanism behind Fkh1’s role in replication regulation, indicating that mere binding of this protein is insufficient for proper regulation of an origin’s firing time.

Our findings are also supported by genome-wide replication initiation data. We observed that the 72 base-pair gap and divergent orientation of Fkh1/2 sites was present at 48 locations throughout the budding yeast genome, with 7 of those overlapping early-firing origins (S1 Table). Relaxing the search criteria by allowing a 71–73 bp gap between the sites increased the number of total hits but did not change the fact that divergent Fkh1/2 consensus sequences preferentially co-localized with early replicating origins (Fig 3A). Combined data from our genome-wide analysis of replication origins revealed that divergently oriented Fkh1/2 consensus sites separated by 71–79 bp overlapped with early origins more frequently than was expected from random distribution of such sites. However, this result may overestimate the tolerance in the gap size between Fkh1/2 sites, given our finding that increasing the gap from 72 bp to 77 bp at *ARS607* results in loss of its ability to bind Fkh1 and to fire early in S phase (Fig 1C and 1D). Overall, our results with modified *ARS607* and *ARS305* indicate that even minor rearrangements of the Fkh1/2 sites are not tolerated and suggest that binding of Fkh1 to replication origins requires precise arrangement of Fkh1/2 binding sites in the locus.

Previous studies have revealed that several sequence elements within *ARS305* and *ARS607* are essential for full activity of these origins. Deletion analysis of *ARS305* demonstrated that the region including the ACS-proximal Fkh1/2 site was required for the origin’s function [14]. Moreover, systematic mutational analysis of *ARS305* identified three short regions, in addition to the ACS, that influenced the stability of plasmids carrying this ARS as a sole replication origin. Mutation of nucleotides immediately adjacent to the 11-bp ACS lead to a complete loss of the origin’s activity, while disruption of either Fkh1/2 site lead to a significant decrease in plasmid retention during exponential cell growth [15]. In addition, yeast DNA replication origins contain nuclease hypersensitive A/T-rich sequences near the ACS, termed DNA unwinding elements [16]. In early origins, these sequences contain one or several continuous stretches of adenine nucleotides (S3 Fig) that may be required for efficient opening of DNA strands by the MCM helicase. Disruption of Fkh1/2 sites or A-tracks within *ARS607* leads to decreased mitotic stability of plasmids, demonstrating the contribution made by these elements to the full activity of the origin [17, 18]. These results support the view that Forkhead proteins enhance replication origins’ efficiency by marking them as ‘first to fire’ when DNA synthesis begins. If Forkhead binding is disturbed, the origin remains functional but loses its early-firing properties and concordantly, becomes less efficient.

Complexity of Fkh1 binding to DNA was further supported by the discovery that formation of the pre-RC at origins was necessary for efficient recruitment of Fkh1. Fkh1 binding to ACS-mutated origins was severely reduced despite the fact that all such loci contained correctly oriented and spaced Fkh1/2 sites (Fig 4B). This suggests either that Fkh1 binding is enhanced by interactions with pre-RC components, or that pre-RC-directed chromatin reorganisation is required for Fkh1 to gain access to the Fkh1/2 consensus sites. Previous studies have shown that replication origins are flanked by strongly positioned nucleosomes and that the ORC complex is needed for this arrangement in vivo and in vitro [19, 20]. Therefore, accessibility of Fkh1/2 sites may be compromised without ORC-dependent nucleosome positioning. On the other hand, one Fkh1/2 site often overlaps the ACS in early origins, suggesting that binding of the ORC and Forkhead are mutually exclusive. However, recent in vitro studies demonstrate that in addition to the ACS, ORC also binds other ACS-like sequences within origins, and that its selectivity towards different binding sites is partially regulated by its interaction with Cdc6 during origin licensing. Moreover, after MCM loading is completed, ORC is removed from the ACS [21, 22]. These results suggest that the ACS is an essential entry point for sequential loading of different pre-RC components onto origins. However, it is not necessarily the final binding site for all recruited proteins complexes. Therefore, redistribution of pre-RC components around the ACS may provide an opportunity for the Forkhead proteins to bind their recognition site within the ACS.

To determine which steps in pre-RC formation are critical for Fkh1 recruitment, we used the origin re-licensing assay [13] that allowed us to monitor re-binding of Fkh1 to the origin in conditions where different pre-RC components were inactivated by temperature-sensitive mutations (Fig 4C). We observed that Fkh1 was not recruited to the origin in *cdc6-ts* or *mcm2-ts* strains, while it was successfully reloaded in a *cdc45-ts* strain (Fig 4E). This indicates that Fkh1 is recruited to origins at the same time or shortly after the pre-RC is fully formed and the Mcm2-7 complex is loaded. This model was further supported by the observation that Fkh1 occupancy at origins decreases significantly in M phase, where origins are not licensed (Fig 4F). By contrast, the next step–recruitment of Cdc45 –is not required for Fkh1 binding. Once recruited to origins, the binding of Fkh1 is presumably stabilised by the ORC complex, as Forkheads interact directly with ORC proteins [4]. However, apparently the ORC alone is not sufficient for efficient recruitment of Forkheads, as the formation of entire pre-RC is required for successful binding of Fkh1 to the origin (Fig 4E). Interestingly, the 71–79 bp gap between Fkh1/2 sites in early origins corresponds very closely to the footprint of the Mcm2-7 double hexamer, which covers about 70–80 bp DNA when loaded onto origins [23, 24]. Therefore, it is possible that on licensed origins the Mcm2-7 complex is stabilised by Forkhead proteins that flank the helicase on both sides. Reciprocally, loading of the Mcm2-7 complex may be necessary to fully expose the Fkh1/2 binding sites. We also noticed that in several early origins one of the Fkh1/2 sites is ‘doubled’–it contains two consensus sequences that overlap partially (S3 Fig). This provides some flexibility of the gap size between Fkh1/2 sites, which might help fine-tune the loading of the pre-RC and Forkhead proteins.

Overall, these results suggest that binding of Fkh1 to replication origins, and possibly to other genomic locations, is a finely regulated process that requires precise arrangement of Fkh1/2 binding sites and the presence of supporting protein complexes in the locus.

## Materials & methods

### Construction of yeast strains

All *Saccharomyces cerevisiae* strains were congenic with strain W303 and are listed in S2 Table. The *GAL-VPS13-ARS* strains contain different versions of *ARS305* and *ARS607* in the *GAL-VPS13* locus, at 3220 bp downstream from the *VPS13* start codon. The following ARS sequences were used for construction of *GAL*-*VPS13*-*ARS* strains (sequence coordinates from the Saccharomyces Genome Database, http://www.yeastgenome.org): *ARS305* (Chr3, nucleotides 39529–39800); *ARS607* (Chr6, nucleotides 199392–199779). To change the distance between Fkh1/2 binding sites in *GAL-VPS13-ARS607*, the distal Fkh1/2 site was mutated (GTAAATA to GATCCTA) and then a new Fkh1/2 site (GTAAATA) was inserted at various distances (10, 30, 60, 90, 120, 150, 180, 240, or 300 bp) away from the ACS-proximal Fkh1/2 site. For finely mapping the tolerance of Fkh binding for altered gap size between Fkh1/2 binding sites within *ARS607*, insertions of 5, 10, or 15 bp were introduced between the two sites in *GAL-VPS13-ARS607* locus. All insertions were located at a distance of 7 bp from the distal Fkh1/2 binding site. 10 bp deletions between the Fkh1/2 sites were made in two different positions in *ARS607*, one located 24 bp and the other 2 bp away from the distal Fkh1/2 site. In *GAL-VPS13-ARS305*, one or both Fkh1/2 consensus binding sequences were reversed (5’ site: TGTTTAT to ATAAACA; 3’ site: GTAAATA to TATTTAC). In strains AKY956 and AKY952, ACS of *GAL-VPS13-ARS305*, or *GAL-VPS13-ARS607* was mutated (in *ARS305*: TTTATATGTTTT to TTTATATGggTT; in *ARS607*: GTTTATATTTAG to GTTTATATccAG). ACS of *ARS305* and *ARS737* (TTTTAATATTT to TTTTAATAccc) were mutated in their native loci in strains AKY1121 and AKY1122, respectively. Sequences of all modified origins are shown in S4 Fig. All modified origins were inserted into genomic loci by two step gene replacement protocol. First, *URA3* gene was inserted into the desired locus and then replaced with ARS sequence by homologous recombination and counter-selection on 5-FOA plates. Strains carrying temperature sensitive alleles *cdc6-1*, *mcm2-td*, or *cdc45-td* [25–27] were used to construct strains AKY1061, AKY1143 and AKY1144 for the origin re-licensing assay. For efficient α-factor arrest, the *BAR1* gene was deleted in all strains.

### Chromatin Immunoprecipitation (ChIP) assay, epitope tags and antibodies

For ChIP assays, the Fkh1 protein was tagged with C-terminal triple E2-tag recognized by a 5E11 antibody (Icosagen), while Mcm4 was tagged with C-terminal triple myc-tag recognized by a 9E10 antibody. Cells were grown in yeast extract-peptone-dextrose (YPD) medium containing 2% glucose as a carbon source before fixation with 1% formaldehyde for the ChIP assay. Cell cycle arrest in G1 was achieved by addition of α-factor-mating pheromone (Zymo Research) to the growth media to a final concentration of 100 nM and by further incubation for 3 hours. Cell cycle arrest in M phase was achieved by incubating the cells with nocadazole (Sigma) with final concentration 20 μg/ml for 60 minutes. ChIP assays were performed as described previously [28]. Shortly, whole-cell extract from 10^7^ cells was used for ChIP assays with 0.5 μg of anti-E2 tag antibody (5E11) or 1 μg of anti myc-tag antibody (9E10). Co-precipitated DNA was analysed by quantitative PCR (qPCR) using Roche Lightcycler 480 real-time PCR system under standard conditions (40 cycles; 95°C for 15 s, 58°C for 20 s, 72°C for 20 s). Maxima SYBR Green/ROX qPCR Master Mix (Thermo Scientific) was used. qPCR was done with primer pairs covering the relevant regions of *VPS13* as well as native origins *ARS607*, *ARS305*, *ARS737* and *ARS522*. Signals were normalized to the high copy-number telomeric *PAU1* gene (in *GAL*-*VPS13*-*ARS607* strains to the native *ARS607* origin). Presented results show the average of three independent experiments, error bars indicate standard deviations. Sequences of qPCR primers are shown in S3 Table.

### DNA replication assay

Yeast strains were arrested in G1 for 3 hours with α-factor and then released into YPD media containing 200mM hydroxyurea (HU) at 24°C. Samples were collected 45 and 75 minutes later by fixing approximately 2x10^7^ cells in 80% ethanol on ice. Subsequently, cells were washed twice with water and disrupted with 0.5mm glass beads in lysis buffer (2% Triton X-100, 1% SDS, 100 mM NaCl, 10 mM Tris-HCl pH 8.0, 1 mM EDTA pH 8.0). Cell lysate was incubated at 40°C for 15 min, genomic DNA was extracted with phenol-chloroform, precipitated with ethanol and dissolved in water. The relative amount of DNA was determined by qPCR with primers specific for *ARS305*, *ARS607*, *ARS522* and *VPS13* loci. The late-replicating locus *ARS522* was used to normalize the data and to calculate the relative increase of the DNA amount at other loci during the experiment. Presented results show the average of three independent experiments, error bars indicate standard deviations.

### Replication origin re-licensing assay

Yeast strains were grown at 24°C in YP-raffinose media for up to 48 hours to obtain culture densities of approximately 1×10^7^ cells/ml. Cells were arrested in G1 for 3 hours with α-factor, then washed once with water and transferred to YP-galactose (containing α-factor) to induce transcription of the *GAL-VPS13-ARS607* cassette. Additionally, during the galactose treatment, temperature was shifted to 37°C to activate the degron system utilized to inactivate Mcm2 or Cdc45 proteins. In order to ensure destruction of Mcm2 and Cdc45, as well as of the temperature-sensitive version of Cdc6, all subsequent steps were carried out at 37°C. Cultures were grown in YP-galactose for 2 hours, following which cells were washed with water and transferred to YPD (pre-warmed to 37°C) containing α-factor. Cultures were incubated at 37°C for 40 minutes, samples were cross-linked with formaldehyde and processed for ChIP analysis. ChIP data were normalized on the native *GAL10* gene, which was regulated by carbon source changes in parallel with the *GAL*-*VPS13*-*ARS607* cassette.

### Analysis of Fkh1/2 binding sites

*Saccharomyces cerevisiae* genome was scanned for single and double Fkh1/2 consensus binding sites (RYMAAYA). For tandem sequences, all possible orientations of the sites: divergent (‘head-to-head’), convergent (‘tail-to-tail’), unidirectional (‘head-to-tail’) were included and gaps of 62 to 88 bp between the sites were allowed. Midpoint coordinates of discovered double Forkhead binding motifs were plotted against the genome-wide dataset of early DNA replication initiation profile (as determined by BrdU incorporation in the presence of HU) [8]. On each chromosome, the highest BrdU signal that did not overlap with any of the confirmed replication origins was set as threshold value. All BrdU peaks over the threshold value were considered to represent genuine early replication origins. Midpoint coordinates of double Forkhead binding sites that were found within 200 bp from BrdU peaks maximum signal were considered as overlapping with the origin. Random overlap was calculated as average overlap between the peaks of early origins and scrambled Fkh1/2 consensus sequences (YAAYMAR, MAARYAY, AAYMYAR) in all orientations, separated by 50–100 bp. All combinations of scrambled sequences were found uniformly over the replication origins with no preference for any particular sequence or orientation (S5 Fig). Late origins were defined as confirmed DNA replication origins in the *S*. *cerevisiae* OriDB (http://cerevisiae.oridb.org) [29] with defined ARS sequence no longer than 600 bp that did not initiate replication in the presence of HU [8]. The analysis of Fkh1/2 consensus site distribution in late origins was performed using the same methodology as for early origins.

## Supporting information

S1 Fig Replication of modified *VPS13*-*ARS607* loci in HU-arrested cells. (PDF)

S2 Fig Replication of modified *VPS13*-*ARS305* loci in HU-arrested cells. (PDF)

S3 Fig Scheme of early origins containing two divergently oriented Fkh1/2 consensus sites. (PDF)

S4 Fig Full sequences of *ARS607* and *ARS305* inserted into *VPS13* locus. (PDF)

S5 Fig Random overlap between scrambled Fkh1/2 sites and replication origins. (PDF)

S1 Table Co-localization of Fkh1/2 patterns with replication origins. (PDF)

S2 Table Yeast strains used in this study. (PDF)

S3 Table qPCR primers used in this study. (PDF)
