# Supplementary material for: Recruitment of Fkh1 to replication origins requires precisely positioned Fkh1/2 binding sites and concurrent assembly of the pre-replicative complex
Source: PLoS Genet. 2017 Jan 31;13(1):e1006588. doi: 10.1371/journal.pgen.1006588 (PMC5308776; doi:10.1371/journal.pgen.1006588)
Supplement: S1 Fig — (PDF) [file pgen.1006588.s001.pdf]

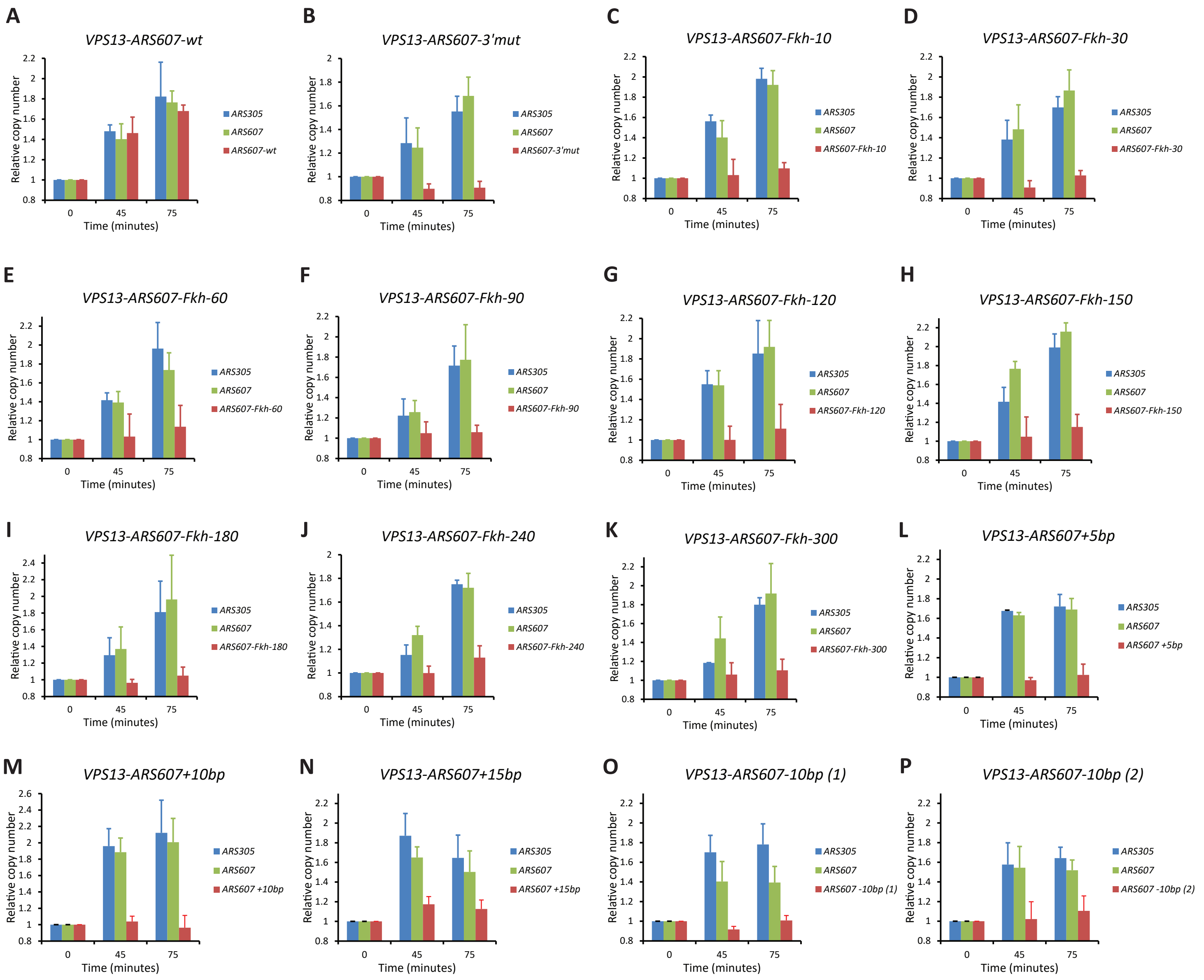

S1 Fig. Each panel represents results from individual strains containing *ARS607-wt* **(A)**, *ARS607-3'mut* **(B)**, *ARS607-Fkh-10* **(C)**, *ARS607-Fkh-30* **(D)**, *ARS607-Fkh-60* **(E)**, *ARS607-Fkh-90* **(F)**, *ARS607-Fkh-120* **(G)**, *ARS607-Fkh-150* **(H)**, *ARS607-Fkh-180* **(I)**, *ARS607-Fkh-240* **(J)**, *ARS607-Fkh-300* **(K),** *ARS607+5bp* **(L)**, *ARS607+10bp* **(M)**, *ARS607+15bp* **(N)**, *ARS607-10bp (1)* **(O)**, or *ARS607-10bp (2)* **(P)** in *VPS13* locus. In each strain native *ARS305* (blue) and *ARS607* (green) loci were used as positive controls to compare the relative DNA copy number in *VPS13-ARS607* loci (red). Cells were first arrested with alpha-factor in G1 and then released into HU-containing media. Total genomic DNA was extracted after 45 and 75 minutes in HU. The amount of DNA from every locus was determined by qPCR and the results are shown as relative to the signal obtained from G1-arrested cells. The ratio between the investigated locus and late-firing *ARS522* in G1-arrested cells (time point 0) was set as 1.
