# Supplementary material for: Recruitment of Fkh1 to replication origins requires precisely positioned Fkh1/2 binding sites and concurrent assembly of the pre-replicative complex
Source: PLoS Genet. 2017 Jan 31;13(1):e1006588. doi: 10.1371/journal.pgen.1006588 (PMC5308776; doi:10.1371/journal.pgen.1006588)
Supplement: S3 Fig — (PDF) [file pgen.1006588.s003.pdf]

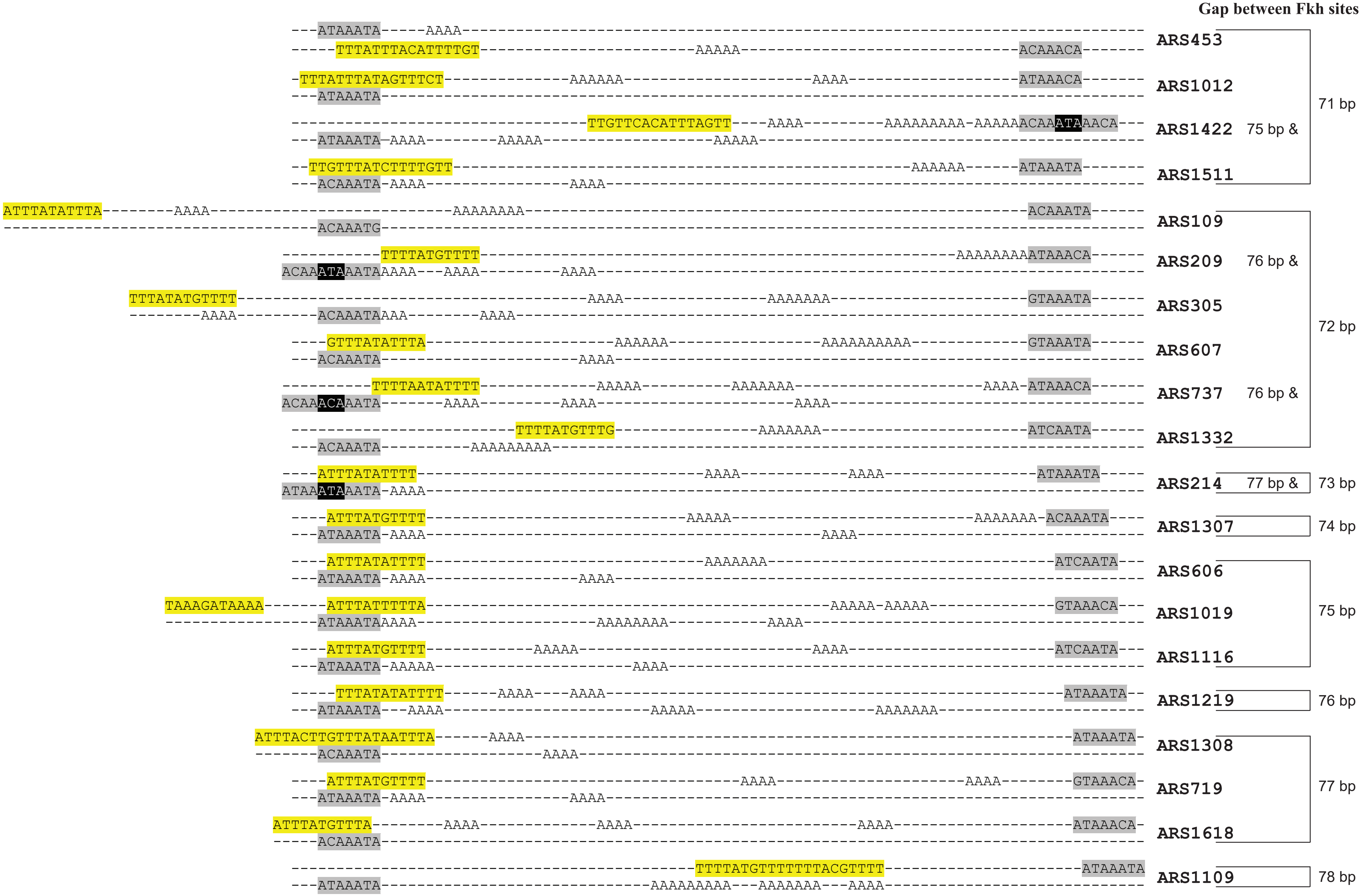

S3 Fig. Scheme of early origins containing two divergently oriented Fkh1/2 consensus sites within 100 bp from ACS. Origins are shown as double stranded DNA with ACS elements (yellow highlighting) and Fkh1/2 consensus sites (gray highlighting). Overlapping region of two adjacent Fkh1/2 consensus sites is marked with black colour. Continuous A-tracks longer than 3 bp are shown with corresponding number of A letters and every other nucleotide is represented as a dash.
