# Supplementary material for: Recruitment of Fkh1 to replication origins requires precisely positioned Fkh1/2 binding sites and concurrent assembly of the pre-replicative complex
Source: PLoS Genet. 2017 Jan 31;13(1):e1006588. doi: 10.1371/journal.pgen.1006588 (PMC5308776; doi:10.1371/journal.pgen.1006588)
Supplement: S4 Fig — (PDF) [file pgen.1006588.s004.pdf]

A

```
Vps13_ARS607_wt       CACTACATTCGCTAAATTACATTATATTATTTCGAGCTTTGTCT
Vps13_ARS607_3' mut   CACTACATTCGCTAAATTACATTATATTATTTCGAGCTTTGTCT
Vps13_ARS607_10       CACTACATTCGCTAAATTACATTATATTATTTCGAGCTTTGTCT
Vps13_ARS607_30       CACTACATTCGCTAAATTACATTATATTATTTCGAGCTTTGTCT
Vps13_ARS607_60       CACTACATTCGCTAAATTACATTATATTATTTCGAGCTTTGTCT
Vps13_ARS607_90       CACTACATTCGCTAAATTACATTATATTATTTCGAGCTTTGTCT
Vps13_ARS607_120      CACTACATTCGCTAAATTACATTATATTATTTCGAGCTTTGTCT
Vps13_ARS607_150      CACTACATTCGCTAAATTACATTATATTATTTCGAGCTTTGTCT
Vps13_ARS607_240      CACTACATTCGCTAAATTACATTATATTATTTCGAGCTTTGTCT
Vps13_ARS607_300      CACTACATTCGCTAAATTACATTATATTATTTCGAGCTTTGTCT
Vps13_ARS607_-10(1)   CACTACATTCGCTAAATTACATTATATTATTTCGAGCTTTGTCT
Vps13_ARS607_-10(2)   CACTACATTCGCTAAATTACATTATATTATTTCGAGCTTTGTCT
Vps13_ARS607_+5       CACTACATTCGCTAAATTACATTATATTATTTCGAGCTTTGTCT
Vps13_ARS607_+10      CACTACATTCGCTAAATTACATTATATTATTTCGAGCTTTGTCT
Vps13_ARS607_+15      CACTACATTCGCTAAATTACATTATATTATTTCGAGCTTTGTCT
Vps13_ARS607_ACS_mut  CACTACATTCGCTAAATTACATTATATTATTTCGAGCTTTGTCT
                      ********************************************

Vps13_ARS607_wt       TGTTTATATTTAGTTAC------------GTTGGGATCAAGTTTTAAAA-----------
Vps13_ARS607_3' mut   TGTTTATATTTAGTTAC------------GTTGGGATCAAGTTTTAAAA-----------
Vps13_ARS607_10       TGTTTATATTTAGTTACGTAAATAAGCTTGTTGGGATCAAGTTTTAAAA-----------
Vps13_ARS607_30       TGTTTATATTTAGTTAC------------GTTGGGATCAAGTTTTAAAAGTAAATAAGCT
Vps13_ARS607_60       TGTTTATATTTAGTTAC------------GTTGGGATCAAGTTTTAAAA-----------
Vps13_ARS607_90       TGTTTATATTTAGTTAC------------GTTGGGATCAAGTTTTAAAA-----------
Vps13_ARS607_120      TGTTTATATTTAGTTAC------------GTTGGGATCAAGTTTTAAAA-----------
Vps13_ARS607_150      TGTTTATATTTAGTTAC------------GTTGGGATCAAGTTTTAAAA-----------
Vps13_ARS607_240      TGTTTATATTTAGTTAC------------GTTGGGATCAAGTTTTAAAA-----------
Vps13_ARS607_300      TGTTTATATTTAGTTAC------------GTTGGGATCAAGTTTTAAAA-----------
Vps13_ARS607_-10(1)   TGTTTATATTTAGTTAC------------GTTGGGATCAAGTTTTAAAA-----------
Vps13_ARS607_-10(2)   TGTTTATATTTAGTTAC------------GTTGGGATCAAGTTTTAAAA-----------
Vps13_ARS607_+5       TGTTTATATTTAGTTAC------------GTTGGGATCAAGTTTTAAAA-----------
Vps13_ARS607_+10      TGTTTATATTTAGTTAC------------GTTGGGATCAAGTTTTAAAA-----------
Vps13_ARS607_+15      TGTTTATATTTAGTTAC------------GTTGGGATCAAGTTTTAAAA-----------
Vps13_ARS607_ACS_mut  TGTTTATATCCAGTTAC------------GTTGGGATCAAGTTTTAAAA-----------
ARS607_ACS sequence    GTTTATATTTA
                      *********..******            ********************

Vps13_ARS607_wt       -AACTAGATCTGGAGTGACCAAAAAAAAAAT------------ACCCA------------
Vps13_ARS607_3' mut   -AACTAGATCTGGAGTGACCAAAAAAAAAAT------------ACCCA------------
Vps13_ARS607_10       -AACTAGATCTGGAGTGACCAAAAAAAAAAT------------ACCCA------------
Vps13_ARS607_30       TAACTAGATCTGGAGTGACCAAAAAAAAAAT------------ACCCA------------
Vps13_ARS607_60       -AACTAGATCTGGAGTGACCAAAAAAAAAATGTAAATAAGCTTACCCA------------
Vps13_ARS607_90       -AACTAGATCTGGAGTGACCAAAAAAAAAAT------------ACCCA------------
Vps13_ARS607_120      -AACTAGATCTGGAGTGACCAAAAAAAAAAT------------ACCCA------------
Vps13_ARS607_150      -AACTAGATCTGGAGTGACCAAAAAAAAAAT------------ACCCA------------
Vps13_ARS607_240      -AACTAGATCTGGAGTGACCAAAAAAAAAAT------------ACCCA------------
Vps13_ARS607_300      -AACTAGATCTGGAGTGACCAAAAAAAAAAT------------ACCCA------------
Vps13_ARS607_-10(1)   -AACTAGAT----------CAAAAAAAAAAT------------ACCCA------------
Vps13_ARS607_-10(2)   -AACTAGATCTGGAGTGACCAAAAAAAAAAT-----------------------------
Vps13_ARS607_+5       -AACTAGATCTGGAGTGACCAAAAAAAAAAT------------ACCCAAGCTT-------
Vps13_ARS607_+10      -AACTAGATCTGGAGTGACCAAAAAAAAAAT------------ACCCAAGCTTACTAG--
Vps13_ARS607_+15      -AACTAGATCTGGAGTGACCAAAAAAAAAAT------------ACCCAAGCTTACTAGAC
Vps13_ARS607_ACS_mut  -AACTAGATCTGGAGTGACCAAAAAAAAAAT------------ACCCA------------
                       ********          ************
```

```
Vps13_ARS607_wt       ---GCGTAAGGTAAATATTATGGATATG------------CTTTGGAAGAAAAGAATTTT
Vps13_ARS607_3’ mut   ---GCGTAAGGATCCTATTATGGATATG------------CTTTGGAAGAAAAGAATTTT
Vps13_ARS607_10       ---GCGTAAGGATCCTATTATGGATATG------------CTTTGGAAGAAAAGAATTTT
Vps13_ARS607_30       ---GCGTAAGGATCCTATTATGGATATG------------CTTTGGAAGAAAAGAATTTT
Vps13_ARS607_60       ---GCGTAAGGATCCTATTATGGATATG------------CTTTGGAAGAAAAGAATTTT
Vps13_ARS607_90       ---GCGTAAGGATCCTATTATGGATATGGTAAATAAGCTTCTTTGGAAGAAAAGAATTTT
Vps13_ARS607_120      ---GCGTAAGGATCCTATTATGGATATG------------CTTTGGAAGAAAAGAATTTT
Vps13_ARS607_150      ---GCGTAAGGATCCTATTATGGATATG------------CTTTGGAAGAAAAGAATTTT
Vps13_ARS607_240      ---GCGTAAGGATCCTATTATGGATATG------------CTTTGGAAGAAAAGAATTTT
Vps13_ARS607_300      ---GCGTAAGGATCCTATTATGGATATG------------CTTTGGAAGAAAAGAATTTT
Vps13_ARS607_-10(1)   ---GCGTAAGGTAAATATTATGGATATG------------CTTTGGAAGAAAAGAATTTT
Vps13_ARS607_-10(2)   --------AGGTAAATATTATGGATATG------------CTTTGGAAGAAAAGAATTTT
Vps13_ARS607_+5       ---GCGTAAGGTAAATATTATGGATATG------------CTTTGGAAGAAAAGAATTTT
Vps13_ARS607_+10      ---GCGTAAGGTAAATATTATGGATATG------------CTTTGGAAGAAAAGAATTTT
Vps13_ARS607_+15      CTAGCGTAAGGTAAATATTATGGATATG------------CTTTGGAAGAAAAGAATTTT
Vps13_ARS607_ACS_mut  ---GCGTAAGGTAAATATTATGGATATG------------CTTTGGAAGAAAAGAATTTT
                              ***    *************            ********************


Vps13_ARS607_wt       GAGACTTACA------------CATTATTCGGCACAGTAGGTACTAGAAAGC--------
Vps13_ARS607_3’ mut   GAGACTTACA------------CATTATTCGGCACAGTAGGTACTAGAAAGC--------
Vps13_ARS607_10       GAGACTTACA------------CATTATTCGGCACAGTAGGTACTAGAAAGC--------
Vps13_ARS607_30       GAGACTTACA------------CATTATTCGGCACAGTAGGTACTAGAAAGC--------
Vps13_ARS607_60       GAGACTTACA------------CATTATTCGGCACAGTAGGTACTAGAAAGC--------
Vps13_ARS607_90       GAGACTTACA------------CATTATTCGGCACAGTAGGTACTAGAAAGC--------
Vps13_ARS607_120      GAGACTTACAGTAAATAAGCTTCATTATTCGGCACAGTAGGTACTAGAAAGC--------
Vps13_ARS607_150      GAGACTTACA------------CATTATTCGGCACAGTAGGTACTAGAAAGCGTAAATAA
Vps13_ARS607_240      GAGACTTACA------------CATTATTCGGCACAGTAGGTACTAGAAAGC--------
Vps13_ARS607_300      GAGACTTACA------------CATTATTCGGCACAGTAGGTACTAGAAAGC--------
Vps13_ARS607_-10(1)   GAGACTTACA------------CATTATTCGGCACAGTAGGTACTAGAAAGC--------
Vps13_ARS607_-10(2)   GAGACTTACA------------CATTATTCGGCACAGTAGGTACTAGAAAGC--------
Vps13_ARS607_+5       GAGACTTACA------------CATTATTCGGCACAGTAGGTACTAGAAAGC--------
Vps13_ARS607_+10      GAGACTTACA------------CATTATTCGGCACAGTAGGTACTAGAAAGC--------
Vps13_ARS607_+15      GAGACTTACA------------CATTATTCGGCACAGTAGGTACTAGAAAGC--------
Vps13_ARS607_ACS_mut  GAGACTTACA------------CATTATTCGGCACAGTAGGTACTAGAAAGC--------
                      **********            ******************************


Vps13_ARS607_wt       ----ATAGAATCACATTCGCCTTTAAAGACATTCTTTTTTCTTGCTCCTTCTATGGACTG
Vps13_ARS607_3’ mut   ----ATAGAATCACATTCGCCTTTAAAGACATTCTTTTTTCTTGCTCCTTCTATGGACTG
Vps13_ARS607_10       ----ATAGAATCACATTCGCCTTTAAAGACATTCTTTTTTCTTGCTCCTTCTATGGACTG
Vps13_ARS607_30       ----ATAGAATCACATTCGCCTTTAAAGACATTCTTTTTTCTTGCTCCTTCTATGGACTG
Vps13_ARS607_60       ----ATAGAATCACATTCGCCTTTAAAGACATTCTTTTTTCTTGCTCCTTCTATGGACTG
Vps13_ARS607_90       ----ATAGAATCACATTCGCCTTTAAAGACATTCTTTTTTCTTGCTCCTTCTATGGACTG
Vps13_ARS607_120      ----ATAGAATCACATTCGCCTTTAAAGACATTCTTTTTTCTTGCTCCTTCTATGGACTG
Vps13_ARS607_150      GCTTATAGAATCACATTCGCCTTTAAAGACATTCTTTTTTCTTGCTCCTTCTATGGACTG
Vps13_ARS607_240      ----ATAGAATCACATTCGCCTTTAAAGACATTCTTTTTTCTTGCTCCTTCTATGGACTG
Vps13_ARS607_300      ----ATAGAATCACATTCGCCTTTAAAGACATTCTTTTTTCTTGCTCCTTCTATGGACTG
Vps13_ARS607_-10(1)   ----ATAGAATCACATTCGCCTTTAAAGACATTCTTTTTTCTTGCTCCTTCTATGGACTG
Vps13_ARS607_-10(2)   ----ATAGAATCACATTCGCCTTTAAAGACATTCTTTTTTCTTGCTCCTTCTATGGACTG
Vps13_ARS607_+5       ----ATAGAATCACATTCGCCTTTAAAGACATTCTTTTTTCTTGCTCCTTCTATGGACTG
Vps13_ARS607_+10      ----ATAGAATCACATTCGCCTTTAAAGACATTCTTTTTTCTTGCTCCTTCTATGGACTG
Vps13_ARS607_+15      ----ATAGAATCACATTCGCCTTTAAAGACATTCTTTTTTCTTGCTCCTTCTATGGACTG
Vps13_ARS607_ACS_mut  ----ATAGAATCACATTCGCCTTTAAAGACATTCTTTTTTCTTGCTCCTTCTATGGACTG
                          ********************************************************
```

```
Vps13_ARS607_wt      CGACACAATAGTTGTTTTTGTGTCGTACCGAAGA------------AAAAGCCTTGTGCA
Vps13_ARS607_3' mut  CGACACAATAGTTGTTTTTGTGTCGTACCGAAGA------------AAAAGCCTTGTGCA
Vps13_ARS607_10      CGACACAATAGTTGTTTTTGTGTCGTACCGAAGA------------AAAAGCCTTGTGCA
Vps13_ARS607_30      CGACACAATAGTTGTTTTTGTGTCGTACCGAAGA------------AAAAGCCTTGTGCA
Vps13_ARS607_60      CGACACAATAGTTGTTTTTGTGTCGTACCGAAGA------------AAAAGCCTTGTGCA
Vps13_ARS607_90      CGACACAATAGTTGTTTTTGTGTCGTACCGAAGA------------AAAAGCCTTGTGCA
Vps13_ARS607_120     CGACACAATAGTTGTTTTTGTGTCGTACCGAAGA------------AAAAGCCTTGTGCA
Vps13_ARS607_150     CGACACAATAGTTGTTTTTGTGTCGTACCGAAGA------------AAAAGCCTTGTGCA
Vps13_ARS607_240     CGACACAATAGTTGTTTTTGTGTCGTACCGAAGAGTAAATAAGCTTAAAAGCCTTGTGCA
Vps13_ARS607_300     CGACACAATAGTTGTTTTTGTGTCGTACCGAAGA------------AAAAGCCTTGTGCA
Vps13_ARS607_-10(1)  CGACACAATAGTTGTTTTTGTGTCGTACCGAAGA------------AAAAGCCTTGTGCA
Vps13_ARS607_-10(2)  CGACACAATAGTTGTTTTTGTGTCGTACCGAAGA------------AAAAGCCTTGTGCA
Vps13_ARS607_+5      CGACACAATAGTTGTTTTTGTGTCGTACCGAAGA------------AAAAGCCTTGTGCA
Vps13_ARS607_+10     CGACACAATAGTTGTTTTTGTGTCGTACCGAAGA------------AAAAGCCTTGTGCA
Vps13_ARS607_+15     CGACACAATAGTTGTTTTTGTGTCGTACCGAAGA------------AAAAGCCTTGTGCA
Vps13_ARS607_ACS_mut CGACACAATAGTTGTTTTTGTGTCGTACCGAAGA------------AAAAGCCTTGTGCA
                     **********************************            **************

Vps13_ARS607_wt      GAAAGCAAAAATAGTACACTTCTAGCCTTGATTACCAAGAGTTATG------------AC
Vps13_ARS607_3' mut  GAAAGCAAAAATAGTACACTTCTAGCCTTGATTACCAAGAGTTATG------------AC
Vps13_ARS607_10      GAAAGCAAAAATAGTACACTTCTAGCCTTGATTACCAAGAGTTATG------------AC
Vps13_ARS607_30      GAAAGCAAAAATAGTACACTTCTAGCCTTGATTACCAAGAGTTATG------------AC
Vps13_ARS607_60      GAAAGCAAAAATAGTACACTTCTAGCCTTGATTACCAAGAGTTATG------------AC
Vps13_ARS607_90      GAAAGCAAAAATAGTACACTTCTAGCCTTGATTACCAAGAGTTATG------------AC
Vps13_ARS607_120     GAAAGCAAAAATAGTACACTTCTAGCCTTGATTACCAAGAGTTATG------------AC
Vps13_ARS607_150     GAAAGCAAAAATAGTACACTTCTAGCCTTGATTACCAAGAGTTATG------------AC
Vps13_ARS607_240     GAAAGCAAAAATAGTACACTTCTAGCCTTGATTACCAAGAGTTATG------------AC
Vps13_ARS607_300     GAAAGCAAAAATAGTACACTTCTAGCCTTGATTACCAAGAGTTATGGTAAATAAGCTTAC
Vps13_ARS607_-10(1)  GAAAGCAAAAATAGTACACTTCTAGCCTTGATTACCAAGAGTTATG------------AC
Vps13_ARS607_-10(2)  GAAAGCAAAAATAGTACACTTCTAGCCTTGATTACCAAGAGTTATG------------AC
Vps13_ARS607_+5      GAAAGCAAAAATAGTACACTTCTAGCCTTGATTACCAAGAGTTATG------------AC
Vps13_ARS607_+10     GAAAGCAAAAATAGTACACTTCTAGCCTTGATTACCAAGAGTTATG------------AC
Vps13_ARS607_+15     GAAAGCAAAAATAGTACACTTCTAGCCTTGATTACCAAGAGTTATG------------AC
Vps13_ARS607_ACS_mut GAAAGCAAAAATAGTACACTTCTAGCCTTGATTACCAAGAGTTATG------------AC
                     **********************************************            **

Vps13_ARS607_wt      AAAAATTTGCAGTTCAGAGTATACCTTAAAAACT
Vps13_ARS607_3' mut  AAAAATTTGCAGTTCAGAGTATACCTTAAAAACT
Vps13_ARS607_10      AAAAATTTGCAGTTCAGAGTATACCTTAAAAACT
Vps13_ARS607_30      AAAAATTTGCAGTTCAGAGTATACCTTAAAAACT
Vps13_ARS607_60      AAAAATTTGCAGTTCAGAGTATACCTTAAAAACT
Vps13_ARS607_90      AAAAATTTGCAGTTCAGAGTATACCTTAAAAACT
Vps13_ARS607_120     AAAAATTTGCAGTTCAGAGTATACCTTAAAAACT
Vps13_ARS607_150     AAAAATTTGCAGTTCAGAGTATACCTTAAAAACT
Vps13_ARS607_240     AAAAATTTGCAGTTCAGAGTATACCTTAAAAACT
Vps13_ARS607_300     AAAAATTTGCAGTTCAGAGTATACCTTAAAAACT
Vps13_ARS607_-10(1)  AAAAATTTGCAGTTCAGAGTATACCTTAAAAACT
Vps13_ARS607_-10(2)  AAAAATTTGCAGTTCAGAGTATACCTTAAAAACT
Vps13_ARS607_+5      AAAAATTTGCAGTTCAGAGTATACCTTAAAAACT
Vps13_ARS607_+10     AAAAATTTGCAGTTCAGAGTATACCTTAAAAACT
Vps13_ARS607_+15     AAAAATTTGCAGTTCAGAGTATACCTTAAAAACT
Vps13_ARS607_ACS_mut AAAAATTTGCAGTTCAGAGTATACCTTAAAAACT
                     **********************************
```

B

```
VPS13_ARS305_3’ rev                                                                  GATTGTATA
VPS13_ARS305_3’ rev                                                                  GATTGTATA
VPS13_ARS305_5’ rev                                                                  GATTGTATA
VPS13_ARS305_5’,3’rev                                                                GATTGTATA
VPS13_ARS305_5’ mut                                                                  GATTGTATA
VPS13_ARS305_ ACS mut                                                                GATTGTATA
                                                                                     *********

VPS13_ARS305 wt         TCGATATATCAGAAACATACATATGATTTTTATCTTGTGAATATTCATCAAACTCCGTTT
VPS13_ARS305_3’ rev     TCGATATATCAGAAACATACATATGATTTTTATCTTGTGAATATTCATCAAACTCCGTTT
VPS13_ARS305_5’ rev     TCGATATATCAGAAACATACATATGATTTTTATCTTGTGAATATTCATCAAACTCCGTTT
VPS13_ARS305_5’,3’ rev  TCGATATATCAGAAACATACATATGATTTTTATCTTGTGAATATTCATCAAACTCCGTTT
VPS13_ARS305_5’ mut     TCGATATATCAGAAACATACATATGATTTTTATCTTGTGAATATTCATCAAACTCCGTTT
VPS13_ARS305_ ACS mut   TCGATATATCAGAAACATACATATGATTTTTATCTTGTGAATATTCATCAAACTCCGTTT
                        ************************************************************

VPS13_ARS305 wt         TTAGCCCCCGTGTAAGTTACTTTAATGAGTATTTGATCCTTTTTTTTTATTGTGTTGGTTT
VPS13_ARS305_3’ rev     TTAGCCCCCGTGTAAGTTACTTTAATGAGTATTTGATCCTTTTTTTTTATTGTGTTGGTTT
VPS13_ARS305_5’ rev     TTAGCCCCCGTGTAAGTTACTTTAATGAGTATTTGATCCTTTTTTTTTATTGTGTTGGTTT
VPS13_ARS305_5’,3’ rev  TTAGCCCCCGTGTAAGTTACTTTAATGAGTATTTGATCCTTTTTTTTTATTGTGTTGGTTT
VPS13_ARS305_5’ mut     TTAGCCCCCGTGTAAGTTACTTTAATGAGTATTTGATCCTTTTTTTTTATTGTGTTGGTTT
VPS13_ARS305_ ACS mut   TTAGCCCCCGTGTAAGTTACTTTAATGAGTATTTGATCCTTTTTTTTTATTGTGTTGGTTT
                        ************************************************************

VPS13_ARS305 wt         TTATATGTTTTGTTATGTATTGTTTATTTTCCCTTTAATTTTAGGATATGAAAACAAGAA
VPS13_ARS305_3’ rev     TTATATGTTTTGTTATGTATTGTTTATTTTCCCTTTAATTTTAGGATATGAAAACAAGAA
VPS13_ARS305_5’ rev     TTATATGTTTTGTTATGTAAATAAACAATTCCCTTTAATTTTAGGATATGAAAACAAGAA
VPS13_ARS305_5’,3’ rev  TTATATGTTTTGTTATGTAAATAAACAATTCCCTTTAATTTTAGGATATGAAAACAAGAA
VPS13_ARS305_5’ mut     TTATATGTTTTGTTATGTATTCTCTATTTTCCCTTTAATTTTAGGATATGAAAACAAGAA
VPS13_ARS305_ACS mut    TTATATGGGTTGTTATGTATTGTTTATTTTCCCTTTAATTTTAGGATATGAAAACAAGAA
ARS 305 ACS sequence     TTATATGTTTT
                        *******  **********         ********************************

VPS13_ARS305 wt         TTTATCAAAGAAAAAAATCTTAGCTTTAAGAACTACAAAGTAAATAAAAAATAATAAATC
VPS13_ARS305_3’ rev     TTTATCAAAGAAAAAAATCTTAGCTTTAAGAACTACAATTATTTACTAAAATAATAAATC
VPS13_ARS305_5’ rev     TTTATCAAAGAAAAAAATCTTAGCTTTAAGAACTACAAAGTAAATAAAAAATAATAAATC
VPS13_ARS305_5’,3’ rev  TTTATCAAAGAAAAAAATCTTAGCTTTAAGAACTACAATTATTTACTAAAATAATAAATC
VPS13_ARS305_5’ mut     TTTATCAAAGAAAAAAATCTTAGCTTTAAGAACTACAAAGTAAATAAAAAATAATAAATC
VPS13_ARS305_ACS mut    TTTATCAAAGAAAAAAATCTTAGCTTTAAGAACTACAAAGTAAATAAAAAATAATAAATC
                        **************************************         *************

VPS13_ARS305 wt         ACACCGGACAGTACATGAAACT
VPS13_ARS305_3’ rev     ACACCGGACAGTACATGAAACT
VPS13_ARS305_5’ rev     ACACCGGACAGTACATGAAACT
VPS13_ARS305_5’,3’ rev  ACACCGGACAGTACATGAAACT
VPS13_ARS305_5’ mut     ACACCGGACAGTACATGAAACT
VPS13_ARS305_ACS mut    ACACCGGACAGTACATGAAACT
                        **********************
```

S4 Fig. Full sequences of *ARS607* **(A)** and *ARS305* **(B)** inserted into *VPS13* locus are shown. Modified or deleted nucleotides are highlighted in red and Fkh1/2 consesnsus sequences are highlighted in turquoise. The inserted sequences are indicated in **bold and underlined**.
