# Supplementary material for: Recruitment of Fkh1 to replication origins requires precisely positioned Fkh1/2 binding sites and concurrent assembly of the pre-replicative complex
Source: PLoS Genet. 2017 Jan 31;13(1):e1006588. doi: 10.1371/journal.pgen.1006588 (PMC5308776; doi:10.1371/journal.pgen.1006588)
Supplement: S5 Fig — (PDF) [file pgen.1006588.s005.pdf]

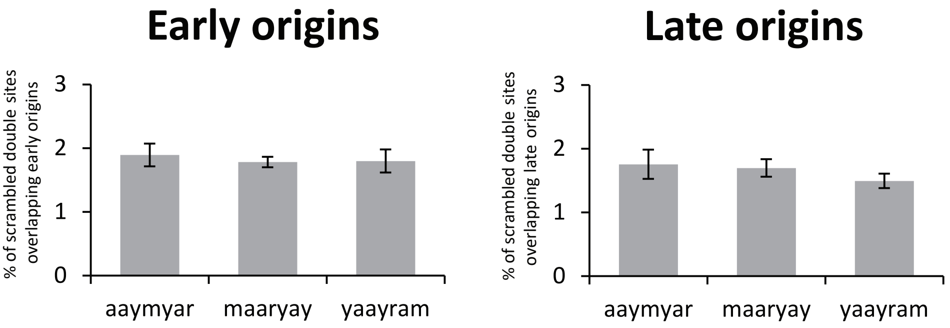

S5 Fig. Random overalap between scrambled Fkh1/2 sites and replication origins. Background was calculated using three scrambled Fkh1/2 consenus sites (AAYMYAR, MAARYAY, YAAYRAM) searched in loci of DNA replication origins with divergent, unidirectional and convergent orientations allowing 50 to 100bp gap between sites. Results show averages of all three orientations with each scrambled consensus.
