# Supplementary material for: Recruitment of Fkh1 to replication origins requires precisely positioned Fkh1/2 binding sites and concurrent assembly of the pre-replicative complex
Source: PLoS Genet. 2017 Jan 31;13(1):e1006588. doi: 10.1371/journal.pgen.1006588 (PMC5308776; doi:10.1371/journal.pgen.1006588)
Supplement: S1 Table — (PDF) [file pgen.1006588.s006.pdf]

S1 Table. Co-localization of Fkh1/2 patterns with early or late firing origins. Genome-wide search results for 81 patterns (two RYMAAYA consensus sequences separated by 62, 63, 64, …88 bp, search was performed for all three orientations) were plotted against genome-wide DNA early replication initiation dataset (Peace et al., 2014) and against confirmed origins that fail replicate in presence of HU. Number of pattern finds and overlaps with early or late origins is shown across the genome for each orientation and per gap size.

Gap	Divergent			Unidirectional			Convergent		
	All hits	Overlap with ARS		All hits	Overlap with ARS		All hits	Overlap with ARS	
		Early	Late		Early	Late		Early	Late
**62**	38	1	2	110	2	4	43	1	0
**63**	43	1	0	86	1	1	45	2	1
**64**	44	1	0	103	3	5	36	0	1
**65**	44	1	1	135	2	3	37	4	0
**66**	55	1	2	89	4	0	33	1	0
**67**	49	3	2	84	0	0	43	1	0
**68**	29	1	0	126	4	3	51	2	1
**69**	30	0	0	87	1	2	55	0	1
**70**	37	2	2	87	2	2	38	3	0
**71**	48	4	0	123	3	2	26	0	0
**72**	48	7	0	86	2	1	71	1	0
**73**	31	3	0	87	1	0	50	1	1
**74**	28	1	1	99	3	3	38	0	0
**75**	42	4	1	84	3	3	34	0	1
**76**	42	3	1	98	3	0	48	1	1
**77**	38	5	0	94	1	0	36	0	2
**78**	46	2	0	78	2	1	44	1	2
**79**	43	1	0	124	5	0	36	0	0
**80**	44	1	1	126	0	1	43	0	1
**81**	57	2	0	90	2	3	38	1	0
**82**	47	1	1	106	10	2	47	2	2
**83**	27	2	1	135	2	0	37	2	0
**84**	36	0	0	128	2	0	73	3	3
**85**	44	3	0	85	4	2	49	2	1
**86**	42	2	0	113	3	0	44	0	0
**87**	31	1	0	93	0	2	41	0	1
**88**	33	0	0	83	2	1	52	1	2
**total**	1096	53	15	2739	67	41	1188	29	21
