# Supplementary material for: Recruitment of Fkh1 to replication origins requires precisely positioned Fkh1/2 binding sites and concurrent assembly of the pre-replicative complex
Source: PLoS Genet. 2017 Jan 31;13(1):e1006588. doi: 10.1371/journal.pgen.1006588 (PMC5308776; doi:10.1371/journal.pgen.1006588)
Supplement: S2 Table — (PDF) [file pgen.1006588.s007.pdf]

S2 Table. Yeast strains used in this study

Strain	Genotype
AKY775	W303, MAT a, *cdc45::CDC45-3x1E2tag-spHIS5, fkh1::FKH1-3x3F12tag-natMX6, bar1Δ::hphMX6*
AKY785	W303, MAT a, *vps13::TRP1-GALprom-vps13_3kb-ARS305-5'-Fkh-mut, cdc45::CDC45-3x1E2tag-spHIS5, fkh1::FKH1-3x3F12tag-natMX6, bar1Δ::hphMX6*
AKY791	W303, MAT a, *vps13::TRP1-GALprom-vps13_3kb-ARS607-wt, cdc45::CDC45-3x1E2tag-spHIS5, fkh1::FKH1-3x3F12tag-natMX6, bar1Δ::hphMX6*
AKY794	W303, MAT a, *vps13::TRP1-GALprom-vps13_3kb-ARS607-3'-Fkh -mut,cdc45::CDC45-3x1E2tag-spHIS5, fkh1::FKH1-3x3F12tag-natMX6, bar1Δ::hphMX6*
AKY901	W303, MAT a, *vps13::TRP1-GALprom-vps13_3kb-ARS607-Fkh10, cdc45::CDC45-3x1E2tag-spHIS5, fkh1::FKH1-3x3F12tag-natMX6, bar1Δ::hphMX6*
AKY902	W303, MAT a, *vps13::TRP1-GALprom-vps13_3kb-ARS607-Fkh30, cdc45::CDC45-3x1E2tag-spHIS5, fkh1::FKH1-3x3F12tag-natMX6, bar1Δ::hphMX6*
AKY903	W303, MAT a, *vps13::TRP1-GALprom-vps13_3kb-ARS607-Fkh60, cdc45::CDC45-3x1E2tag-spHIS5, fkh1::FKH1-3x3F12tag-natMX6, bar1Δ::hphMX6*
AKY904	W303, MAT a, *vps13::TRP1-GALprom-vps13_3kb-ARS607-Fkh90, cdc45::CDC45-3x1E2tag-spHIS5, fkh1::FKH1-3x3F12tag-natMX6, bar1Δ::hphMX6*
AKY905	W303, MAT a, *vps13::TRP1-GALprom-vps13_3kb-ARS607-Fkh120, cdc45::CDC45-3x1E2tag-spHIS5, fkh1::FKH1-3x3F12tag-natMX6, bar1Δ::hphMX6*
AKY906	W303, MAT a, *vps13::TRP1-GALprom-vps13_3kb-ARS607-Fkh150, cdc45::CDC45-3x1E2tag-spHIS5, fkh1::FKH1-3x3F12tag-natMX6, bar1Δ::hphMX6*
AKY907	W303, MAT a, *vps13::TRP1-GALprom-vps13_3kb-ARS607-Fkh180, cdc45::CDC45-3x1E2tag-spHIS5, fkh1::FKH1-3x3F12tag-natMX6, bar1Δ::hphMX6*
AKY908	W303, MAT a, *vps13::TRP1-GALprom-vps13_3kb-ARS607-Fkh240, cdc45::CDC45-3x1E2tag-spHIS5, fkh1::FKH1-3x3F12tag-natMX6, bar1Δ::hphMX6*
AKY909	W303, MAT a, *vps13::TRP1-GALprom-vps13_3kb-ARS607-Fkh300, cdc45::CDC45-3x1E2tag-spHIS5, fkh1::FKH1-3x3F12tag-natMX6, bar1Δ::hphMX6*
AKY930	W303, MAT a, *cdc45::CDC45-3x1E2tag-spHIS5, fkh1::FKH1-3x3F12tag-natMX6, mcm4::MCM4-3xMyc tag-LEU2, bar1Δ::hphMX6*
AKY946	W303, MAT a, *vps13::TRP1-GALprom-vps13_3kb-ARS607-wt, cdc45::CDC45-3x1E2tag-spHIS5, fkh1::FKH1-3x3F12tag-natMX6, mcm4::MCM4-3xMyc tag-LEU2, bar1Δ::hphMX6*
AKY948	W303, MAT a, *vps13::TRP1-GALprom-vps13_3kb-ARS607-wt, orc2::ORC2-3x1E2tag-spHIS5, fkh1::FKH1-3x3F12tag-natMX6, mcm4::MCM4-3xMyc tag-LEU2, bar1Δ::hphMX6*
AKY952	W303, MAT a, *vps13::TRP1-GALprom-vps13_3kb-ARS607-ACSmut, cdc45::CDC45-3x1E2tag-spHIS5, fkh1::FKH1-3x3F12tag-natMX6, bar1Δ::hphMX6*

AKY953	W303, MAT a, *vps13::TRP1-GALprom-vps13_3kb-ARS305-5'-3'-Fkh-rev, cdc45::CDC45-3x1E2tag-spHIS5, fkh1::FKH1-3x3F12tag-natMX6, bar1Δ::hphMX6*
AKY954	W303, MAT a, *vps13::TRP1-GALprom-vps13_3kb-ARS305-3'-Fkh-rev, cdc45::CDC45-3x1E2tag-spHIS5, fkh1::FKH1-3x3F12tag-natMX6, bar1Δ::hphMX6*
AKY955	W303, MAT a, *vps13::TRP1-GALprom-vps13_3kb-ARS305-5'-Fkh-rev, cdc45::CDC45-3x1E2tag-spHIS5, fkh1::FKH1-3x3F12tag-natMX6, bar1Δ::hphMX6*
AKY956	W303, MAT a, *vps13::TRP1-GALprom-vps13_3kb-ARS305-ACSmut, cdc45::CDC45-3x1E2tag-spHIS5, fkh1::FKH1-3x3F12tag-natMX6, bar1Δ::hphMX6*
AKY992	W303, MAT a, *vps13::TRP1-GALprom-vps13_3kb-ARS305-wt, cdc45::CDC45-3x1E2tag-spHIS5, fkh1::FKH1-3x3F12tag-natMX6, bar1Δ::hphMX6*
AKY1061	W303, MAT a, *cdc6-1, vps13::TRP1-GALprom-vps13_3kb-ARS607-wt, orc2::ORC2-3x1E2tag-spHIS5, fkh1::FKH1-3x3F12tag-natMX6, mcm4::MCM4-3xMyc tag-LEU2, bar1Δ::hphMX6*
AKY1121	W303, MAT a, *ars305::ARS305-ACSmut, cdc45::CDC45-3x1E2tag-spHIS5, fkh1::FKH1-3x3F12tag-natMX6, mcm4::MCM4-3xMyc tag-LEU2, bar1Δ::hphMX6*
AKY1122	W303, MAT a, *ars737::ARS737-ACSmut, cdc45::CDC45-3x1E2tag-spHIS5, fkh1::FKH1-3x3F12tag-natMX6, mcm4::MCM4-3xMyc tag-LEU2, bar1Δ::hphMX6*
AKY1143	W303, MAT a, *mcm2::mcm2-td-URA3, ubr1::GAL-Ub-Myc-UBR1-spHIS5, vps13::TRP-GALprom-vps13_3kb-ARS607-wt, orc2::ORC2-3x1E2tag-kanMX, fkh1::FKH1-3x3F12tag-natMX6, mcm4::MCM4-3xFLAG tag-LEU2, bar1Δ::hphMX6*
AKY1144	W303, MAT a, *cdc45::cdc-td-TRP1, ubr1::GAL-Ub-Myc-UBR1-spHIS5, vps13::TRP-GALprom-vps13_3kb-ARS607-wt, orc2::ORC2-3x1E2tag-kanMX, fkh1::FKH1-3x3F12tag-natMX6, mcm4::MCM4-3xFLAG tag-LEU2, bar1Δ::hphMX6*
AKY1301	W303, MAT a, *vps13::TRP1-GALprom-vps13_3kb-ARS607-Fkh10, cdc45::CDC45-3x1E2tag-spHIS5, fkh1::FKH1-3x3F12tag-natMX6, mcm4::MCM4-3xMyc tag-LEU2, bar1Δ::hphMX6*
AKY1302	W303, MAT a, *vps13::TRP1-GALprom-vps13_3kb-ARS607-Fkh30, cdc45::CDC45-3x1E2tag-spHIS5, fkh1::FKH1-3x3F12tag-natMX6, mcm4::MCM4-3xMyc tag-LEU2, bar1Δ::hphMX6*
AKY1303	W303, MAT a, *vps13::TRP1-GALprom-vps13_3kb-ARS607-Fkh60, cdc45::CDC45-3x1E2tag-spHIS5, fkh1::FKH1-3x3F12tag-natMX6, mcm4::MCM4-3xMyc tag-LEU2, bar1Δ::hphMX6*
AKY1304	W303, MAT a, *vps13::TRP1-GALprom-vps13_3kb-ARS607-Fkh90, cdc45::CDC45-3x1E2tag-spHIS5, fkh1::FKH1-3x3F12tag-natMX6, mcm4::MCM4-3xMyc tag-LEU2, bar1Δ::hphMX6*
AKY1305	W303, MAT a, *vps13::TRP1-GALprom-vps13_3kb-ARS607-Fkh120, cdc45::CDC45-3x1E2tag-spHIS5, fkh1::FKH1-3x3F12tag-natMX6, mcm4::MCM4-3xMyc tag-LEU2, bar1Δ::hphMX6*
AKY1306	W303, MAT a, *vps13::TRP1-GALprom-vps13_3kb-ARS607-Fkh150, cdc45::CDC45-3x1E2tag-spHIS5, fkh1::FKH1-3x3F12tag-natMX6, mcm4::MCM4-3xMyc tag-LEU2, bar1Δ::hphMX6*
AKY1307	W303, MAT a, *vps13::TRP1-GALprom-vps13_3kb-ARS607-Fkh180, cdc45::CDC45-3x1E2tag-spHIS5, fkh1::FKH1-3x3F12tag-natMX6, mcm4::MCM4-3xMyc tag-LEU2, bar1Δ::hphMX6*

AKY1308	W303, MAT a, *vps13::TRP1-GALprom-vps13_3kb-ARS607-Fkh240, cdc45::CDC45-3x1E2tag-spHIS5, fkh1::FKH1-3x3F12tag-natMX6, mcm4::MCM4-3xMyc tag-LEU2, bar1Δ::hphMX6*
AKY1309	W303, MAT a, *vps13::TRP1-GALprom-vps13_3kb-ARS607-Fkh300, cdc45::CDC45-3x1E2tag-spHIS5, fkh1::FKH1-3x3F12tag-natMX6, mcm4::MCM4-3xMyc tag-LEU2, bar1Δ::hphMX6*
AKY1310	W303, MAT a, *vps13::TRP1-GALprom-vps13_3kb-ARS607-3'-Fkh-mut, cdc45::CDC45-3x1E2tag-spHIS5, fkh1::FKH1-3x3F12tag-natMX6, mcm4::MCM4-3xMyc tag-LEU2, bar1Δ::hphMX6*
AKY1311	W303, MAT a, *vps13::TRP1-GALprom-vps13_3kb-ARS607-ACSmut, cdc45::CDC45-3x1E2tag-spHIS5, fkh1::FKH1-3x3F12tag-natMX6, mcm4::MCM4-3xMyc tag-LEU2, bar1Δ::hphMX6*
AKY1312	W303, MAT a, *vps13::TRP1-GALprom-vps13_3kb-ARS305-wt, cdc45::CDC45-3x1E2tag-spHIS5, fkh1::FKH1-3x3F12tag-natMX6, mcm4::MCM4-3xMyc tag-LEU2, bar1Δ::hphMX6*
AKY1313	W303, MAT a, *vps13::TRP1-GALprom-vps13_3kb-ARS305-5'-3'-Fkh-rev, cdc45::CDC45-3x1E2tag-spHIS5, fkh1::FKH1-3x3F12tag-natMX6, mcm4::MCM4-3xMyc tag-LEU2, bar1Δ::hphMX6*
AKY1314	W303, MAT a, *vps13::TRP1-GALprom-vps13_3kb-ARS305-5'-Fkh-rev, cdc45::CDC45-3x1E2tag-spHIS5, fkh1::FKH1-3x3F12tag-natMX6, mcm4::MCM4-3xMyc tag-LEU2, bar1Δ::hphMX6*
AKY1315	W303, MAT a, *vps13::TRP1-GALprom-vps13_3kb-ARS305-3'-Fkh-rev, cdc45::CDC45-3x1E2tag-spHIS5, fkh1::FKH1-3x3F12tag-natMX6, mcm4::MCM4-3xMyc tag-LEU2, bar1Δ::hphMX6*
AKY1524	W303, MAT a, *vps13::TRP1-GALprom-vps13_3kb-ARS607-5bp-insert, cdc45::CDC45-3x1E2tag-spHIS5, fkh1::FKH1-3x3F12tag-natMX6, mcm4::MCM4-3xMyc tag-LEU2, bar1Δ::hphMX6*
AKY1525	W303, MAT a, *vps13::TRP1-GALprom-vps13_3kb-ARS607-10bp-insert, cdc45::CDC45-3x1E2tag-spHIS5, fkh1::FKH1-3x3F12tag-natMX6, mcm4::MCM4-3xMyc tag-LEU2, bar1Δ::hphMX6*
AKY1526	W303, MAT a, *vps13::TRP1-GALprom-vps13_3kb-ARS607-15bp-insert, cdc45::CDC45-3x1E2tag-spHIS5, fkh1::FKH1-3x3F12tag-natMX6, mcm4::MCM4-3xMyc tag-LEU2, bar1Δ::hphMX6*
AKY1527	W303, MAT a, *vps13::TRP1-GALprom-vps13_3kb-ARS607-10bp-del1, cdc45::CDC45-3x1E2tag-spHIS5, fkh1::FKH1-3x3F12tag-natMX6, mcm4::MCM4-3xMyc tag-LEU2, bar1Δ::hphMX6*
AKY1528	W303, MAT a, *vps13::TRP1-GALprom-vps13_3kb-ARS607-10bp-del2, cdc45::CDC45-3x1E2tag-spHIS5, fkh1::FKH1-3x3F12tag-natMX6, mcm4::MCM4-3xMyc tag-LEU2, bar1Δ::hphMX6*
