# Supplementary material for: Recruitment of Fkh1 to replication origins requires precisely positioned Fkh1/2 binding sites and concurrent assembly of the pre-replicative complex
Source: PLoS Genet. 2017 Jan 31;13(1):e1006588. doi: 10.1371/journal.pgen.1006588 (PMC5308776; doi:10.1371/journal.pgen.1006588)
Supplement: S3 Table — (PDF) [file pgen.1006588.s008.pdf]

S3 Table. qPCR primers used in this study

**ARS305**	Forward	5’- GCAGTGCTTGTAACTGGTGC
	Reverse	5’- TAGTTATTACGGCGTCGGGC
**ARS522**	Forward	5’- ATTGAGCATTACCTAACGCCATA
	Reverse	5’- TGGATCTGAAACCGAGCAGTT
**ARS607**	Forward	5’- GGCTCGTGCATTAAGCTTGTA
	Reverse	5’- CAATAGCAGGATCGACCTGACT
**ARS737**	Forward	5’- TGCTTATTAAGGGTCTAGGACATTT
	Reverse	5’- ACTTTTGCTTAAGCGGCAGAAT
**VPS13-3kb**	Forward	5’- TGATTCTATAAAGCTGGCAACGT
	Reverse	5’- CTAAATACCGAATCCCTGGAAAA
**PAU1**	Forward	5’- TGGCCCAATACTACATGTTCCA
	Reverse	5’- GTAGTTGAAAACGGCTTCAGCA
**GAL10**	Forward	5’- CTTAGGGCCTACTAATCCGTAT
	Reverse	5’- TTGTTTGGTATACCTAGCGGAT
